# Experimental Highly Pathogenic Avian Influenza A(H5N1) Clade 2.3.4.4b Virus Infection in Alpacas, 2026

**DOI:** 10.3201/eid3210.260491

**Published:** 2026-10

**Authors:** Jacob Schön, Angele Breithaupt, Nico Joel Halwe, Maxi Hertel, Ann Kathrin Ahrens, Andrea Aebischer, Donata Hoffmann, Martin Beer

**Affiliations:** Institute of Diagnostic Virology, Friedrich-Loeffler-Institut, Greifswald, Germany (J. Schön, N.J. Halwe, A.K. Ahrens, A. Aebischer, D. Hoffman, M. Beer); Department of Experimental Animal Facilities and Biorisk Management, Friedrich-Loeffler-Institut, Greifswald (A. Breithaupt, M. Hertel)

**Keywords:** Influenza, respiratory infections, viruses, H5N1, highly pathogenetic avian influenza, HPAI, clade 2.3.4.4b, B3.13, alpacas, Germany

## Abstract

Highly pathogenic avian influenza (HPAI) A(H5N1) clade 2.3.4.4b virus continues to spread globally and sporadically transmits from avian reservoirs to mammalian hosts. In May 2024, H5N1 infections in young goats and alpacas in the United States were reported. Nevertheless, the overall susceptibility of camelids to clade 2.3.4.4b virus remains unclear. We conducted a controlled experimental infection study in 6 alpacas, assessing clinical signs, viral shedding, tissue distribution, and serologic responses after intranasal inoculation with HPAI H5N1 genotype B3.13 virus. Observed illness was generally mild; body temperature increased slightly and food intake reduced for up to 3 days postinfection. We detected viral RNA in nasal swab samples and confirmed infectious HPAI H5N1 virus. Immunohistochemistry and RNA in situ hybridization detected virus only in the nasopharyngeal tonsil and nasal conchae at 4 days postinfection. Our findings suggest alpacas are susceptible to productive H5N1 infection, highlighting implications for livestock surveillance and biosecurity in regions with ongoing circulation.

In 1996, highly pathogenic avian influenza (HPAI) H5N1 virus (goose/Guangdong/96) appeared in southern China, causing outbreaks in poultry and infecting 18 humans, 6 of whom died (*1*–*3*). Descendants of that lineage subsequently triggered major outbreaks in poultry in Southeast Asia and Europe, including H5N1 clades 2.2 (2005–2006) and 2.3.2.1c (2010) and H5N8 clade 2.3.4.4a (2014), reflecting a global rise in HPAI virus (HPAIV) outbreaks (*4*–*7*). In 2016, Europe experienced major HPAI H5 clade 2.3.4.4b outbreaks in wild birds and domestic poultry (*4*), followed by major outbreaks during 2020–2021 (*7**–**9*); the clade then became endemic (*10*). Nevertheless, during that period, clade 2.3.4.4b virus continuously evolved; serotype H5N8 predominated in 2016, transitioning to H5N6 by 2018, and shifting to H5N1 by 2020 (*9*,*11*,*12*). Although the virus remained in the same clade as classified by homologies of the hemagglutinin (HA) segment, the genotypes changed through continuous reassortment events (*7*,*13*). 

In December 2021, HPAI H5N1 clade 2.3.4.4b virus spread from Europe to Canada and the United States via wild birds (*14*,*15*). Subsequently, several Eurasian H5N1 clade 2.3.4.4b virus genotypes entered North America, and lineages A1 and A3 reassorted with local low pathogenicity avian influenza viruses in migratory birds, giving rise to new H5N1 clade 2.3.4.4b genotypes like B3.13 (late 2023–early 2024) and D1.1 (September 2024) (*15*,*16*). Those viruses spread rapidly in US wild bird populations, including waterfowl, birds of prey, seabirds, shorebirds, and a wide range of common land birds; during December 2021–December 2025, a total of 17,718 wild birds tested positive (*17*). In addition, clade 2.3.4.4b virus spread to domestic livestock birds, affecting 186.18 million birds in 2,029 flocks during February 2022–January 2026 (*18*). 

Besides avian species, a wide range of mammals have been infected, including 683 wild mammals tested during April 2022–December 2025, mostly terrestrial carnivores feeding on H5-infected birds, including 148 red fox (*Vulpus vulpus*), 81 skunks (various species), 34 raccoons (*Procyon lotor*), 28 mountain lions (*Puma concolor*), and 18 bobcats (*Lynx rufus*) (*19*). In addition, 148 domestic cats (*Felis catus*), 110 house mice (*Mus musculus*), and 26 deer mice (*Peromyscus* spp.) also were infected, mostly in the context of affected farms (*19*). 

In March 2024, H5N1 clade 2.3.4.4b genotype B3.13 virus was detected in US dairy cattle, representing the first well-documented instance of widespread HPAIV infection in bovines (*20*). That detection was accompanied by evidence of high infectious virus shedding in milk, making milk-mediated cow-to-cow transmission highly likely (*20*–*23*). Concurrently, young goat kids co-located with infected poultry on a Minnesota farm exhibited neurologic signs and tested positive for H5N1 genotype B3.6 virus (*24*,*25*). During March 2024–May 2025, a total of 70 cases of human H5N1 infection were reported in the United States (*26*); most were associated with exposure to infected dairy cattle (41 cases) or commercial poultry (24 cases) (*15*). Among human cases, the most common reported symptoms were conjunctivitis (89%), fever (46%), and respiratory symptoms (41%) (*26*). All sequenced viruses from human cases belonged to clade 2.3.4.4b, predominantly B3.13 and D1.1 genotypes, but 1 D1.3 genotype virus also was identified (*15*).

In May 2024, the US National Veterinary Services Laboratories confirmed detection of HPAI H5N1 virus from alpacas at a premises where poultry had previously tested positive (*27*). The viral genome matched the B3.13 genotype circulating in US dairy cattle and the depopulated poultry, strongly implicating environmental contamination and cross-species exposure (*27*). The alpacas shared pasture and water sources with infected poultry and ducks but initially showed no clinical signs (*27**–**29*). However, on May 10, the day H5N1 virus infection was confirmed in poultry, 1 alpaca experienced spontaneous late-term abortion; 2 more aborted on May 14 and 15, and another spontaneous abortion occurred before September 2024 (*29*). By May 15, 2024, four of 18 alpacas showed clinical signs, including depression, weakness, and mild respiratory symptoms (*29*). An additional alpaca died acutely, without prior reported signs, 10 days after the first reports of influenza-like illness in poultry; a nasal swab sample collected on the day of death tested H5N1-positive, but no postmortem examination was conducted (*29*). Of the 18 adult alpacas (14 female, 4 male), only the female animals were affected (*29*). One aborted fetus was later examined, and H5N1 was confirmed in brain tissue (*29*). Ten surviving female alpacas seroconverted (*29*). Of note, alpaca milk samples tested positive for viral genome, indicating acute infection of the udder, but also later tested positive for influenza virus A–specific antibodies (*29*). On the same farm, milk from 1 llama tested positive for specific antibodies 4 months after the outbreak, and 2 of 13 yaks (both female) also seroconverted (*29*). That detection expanded the known host range of HPAI H5N1 virus and raised questions regarding its susceptibility, pathogenesis, and transmission dynamics in camelids. 

HPAIV H5N1 continues to pose a threat to birds and, under certain conditions, to mammalian species. Although spillover into mammals is generally rare, the recent detections in nonavian hosts have heightened concerns about cross-species transmission, adaptation, and the emergence of novel mammalian hosts (*11*,*20*). Given that precedent, controlled experimental infection studies can address key knowledge gaps, including the incubation period, clinical spectrum, transmission routes, duration of viral shedding, tissue tropism, and the potential for adaptation in nonavian hosts. We report an experimental challenge study in alpacas using HPAIV H5N1 genotype B3.13, the same genotype identified in the US field case in camelids, to characterize virus kinetics, pathology, and shedding. Our aim was to clarify risk for H5N1 infection among camelid populations, elucidate cross-species transmission dynamics, and better define the potential for additional mammalian adaptation.

## Methods

The experiment and all subsequent sample analyses were conducted at the Friedrich-Loeffler-Institut (Greifswald, Germany) in the licensed high containment Biosafety Level 3 laboratories and Biosafety Level 3 large animal facilities by using required personal protective measures in accordance with the respective permits. We obtained 6 healthy male alpacas (*Vicugna pacos*), 12–24 months of age, from a local breeder. We housed animals in 2 groups of 3 (stable 1 and stable 2) and allowed them to acclimatize to housing conditions for >2 weeks before infection. We provided loose hay in hay bags and specialized camelid feed in troughs; water was available ad libitum throughout the study period.

### Experimental Setup and Sampling

We obtained baseline jugular venous blood samples and nasal and rectal swab samples 1 day before inoculation. Then, we intranasally inoculated each of the 6 alpacas by using a nasal nebulizer device on a 1-mL syringe. Animals received 10^5.9^ 50% tissue culture infectious dose (TCID_50_), 10^5.6^ TCID_50_/mL per nostril, of HPAI H5N1 genotype B3.13 virus isolate A/Cattle/Texas/063224-24-1/2024 (GISAID accession no. EPI_ISL_19155861). We confirmed inoculum titer by back-titration. 

Staff monitored animals daily for wellbeing and collected nasal and rectal swab samples on days 1–9 and 11 after inoculation. At 4 days postinoculation (dpi), the 3 animals from stable 1 were euthanized to collect serum and organ samples, including nasal conchae (respiratory and olfactory regions), trachea (cranial and caudal), lungs (all lobes), liver, kidney, and spleen. The 3 animals from stable 2 were euthanized at 20 dpi for collection of the same sample panel.

### Sample Preparation

We collected blood samples by using KABEVETTE G systems with disposable needles (KABE Labortechnik, https://kabe-labortechnik.de). We suspended swabs in 2 mL of Dulbecco Modified Eagle Medium supplemented with 1 mg/mL enrofloxacin, 1 mg/mL lincomycin, 0.05 mg/mL gentamycin, and 0.05 mg/mL amphotericin. We transferred organ samples (≈2 mm^3^) into tubes containing medium with penicillin (10 U/mL) and streptomycin (10 µg/mL) and a stainless-steel bead. We homogenized organ samples for 2 minutes at 300 Hz by using TissueLyser II (QIAGEN, https://www.qiagen.com). We extracted viral RNA from 100 µL of swab medium and organ homogenates by using the NucleoMag Vet kit (Macherey-Nagel, https://www.mn-net.com) on a BioSprint 96 platform (QIAGEN).

### ELISAs

We evaluated serum samples by using 2 different ELISAs. First, we used an in-house clade 2.3.4.4b–adapted H5 nanoluciferase–based ELISA (*30*). In brief, we coated high-binding ELISA plates overnight at 4°C with 0.2 µg/well H5 HA1 and blocked with 5% skim milk. We preincubated 1:10 serum samples with 2 × 10^5^ luciferase light units (LU) H5-nanoluciferase for 1 hour at room temperature, then added to ELISA plates for and incubated another hour. We measured bioluminescence in LU by using the Nano-Glo Luciferase Assay System (Promega, https://www.promega.com). Second, we used an updated version of the ID Screen Influenza H5 Antibody Competition 3.0 Multi-species ELISA (Innovative Diagnostics, https://innovative-diagnostics.com), following the manufacturer’s protocol. We diluted heat-inactivated serum 1:10 and incubated on H5-coated plates with controls. After washing, we sequentially added conjugate and substrate and measured absorbance at 450 nm. We interpreted results by using bovine thresholds, considering signal-to-noise ratio <40% as positive and 40%–50% as doubtful. 

### Virus Neutralization Test

We determined virus-neutralizing antibodies by using a 100% virus neutralization assay on MDCKII cells. We serially diluted serum samples by log_2_ steps, mixed with 100 TCID_50_ of virus, and incubated for 1 hour at 37°C. We then added MDCKII cells and incubated at 37°C for 72 hours. We defined neutralization titers as the highest dilution without visible cytopathic effect.

### Viral RNA Detection

We used quantitative reverse transcription PCR (qRT-PCR) to detect matrix (M) gene segment 7 of the H5N1 virus genome (*31*), according to European Union Reference Laboratory recommendations (*32*). We performed relative quantification in CFX Maestro software (Bio-Rad Laboratories, https://www.bio-rad.com).

### Infectious Titer Determination

We determined virus titers in nasal swab samples and PCR-positive organ samples by TCID_50_ endpoint dilution assays on MDCKII cells. We applied serial 10-fold dilutions to cell monolayers and incubated at 37°C for 72 hours, then calculated titers by using the Spearman–Kärber method on the basis of cytopathic effect.

### Pathology

During necropsies, we recorded lesions and fixed tissue samples in 4% buffered formaldehyde. On the basis of virologic data, we paraffin-embedded nasal conchae from the respiratory and olfactory regions, nasopharyngeal tonsil, mandibular lymph node, trachea (cranial, caudal), lung (left cranial, caudal lobe; right cranial, middle, caudal, accessory lobe), and brain. We sectioned 2–4 μm of tissue and stained with hematoxylin and eosin, following routine protocols (*33*). We prepared consecutive sections for viral antigen detection by using a primary antibody against influenza A virus (IAV) nucleoprotein (ATCC clone HB-64), as previously described (*21*). As a negative control, we tested 20 dpi PCR-negative conchae with the primary antibody and labeled consecutive sections of PCR-positive samples with a 1:45 dilution of irrelevant SARS antibody clone 4F3C4 (*21*). We processed consecutive slides of the nasal conchae and tonsil for RNA in situ hybridization (ISH). To visualize RNA, we used a custom-designed probe and the RNAScope 2-5 HD Reagent Kit Red (Advanced Cell Diagnostics, https://info.acdbio.com), according to the manufacturer’s instructions. We used probes against peptidylprolyl isomerase B and dihydrodipicolinate reductase genes as technical controls. We used archived tissues from HPAIV-infected chickens as positive controls for immunohistochemistry (IHC) and ISH. 

To clarify histologic findings, we used IHC and ISH to test the olfactory bulb of animal 6 for virus antigen and RNA, as described. We performed immune cell labeling by using a 1:500 dilution of Anti Iba-1, Rabbit (FUJIFILM Wako Chemicals Europe, https://labchem-wako.fujifilm.com) antibodies against ionized calcium binding adaptor molecule 1 (Iba-1) targeting microglia and macrophages. To detect Iba-1 antibodies, we used biotinylated goat anti-rabbit IgG secondary antibody and incubated slides with Vectastain Elite ABC-HRP Kit (both Vector Laboratories, https://vectorlabs.com). We also targeted T cells by using a 1:100 dilution of Polyclonal Rabbit Anti-Human CD3 (DAKO Agilent, https://www.agilent.com) and ImmPRESS HRP Goat Anti-Rabbit IgG Polymer Detection Kit (Vector Laboratories) for detection. We used sections of the nasal tonsil as positive controls for Iba-1 and CD3. As a negative control, we tested consecutive sections with an irrelevant antibody targeting rustrela virus clone 2H11B1. We produced bright red intracytoplasmic chromogen labeling by using the AEC Substrate Kit (abcam, https://www.abcam.com). We counter-stained sections with Mayer hematoxylin.

A pathologist certified through the European College of Veterinary Pathologists digitally evaluated and interpreted IHC and ISH by using NDP.view2 software (Hamamatsu Photonics, https://www.hamamatsu.com). We also processed brain paraffin sections for viral genome detection by PCR following the same protocol as other tissues.

## Results

### Clinical Signs

We observed no major reduction in alpaca wellbeing after H5N1 infection. Overall mean body temperature remained constant (38.3°C) and within physiologic range (37.5°C–38.9°C) (*34*), but animals 3 and 5 had temperature peaks of 39.6°C at 1 dpi, and animal 2’s temperature peaked at 39.1°C at 2 dpi, indicating early antiviral immune response (Figure 1, panel A). 

**Figure 1:**
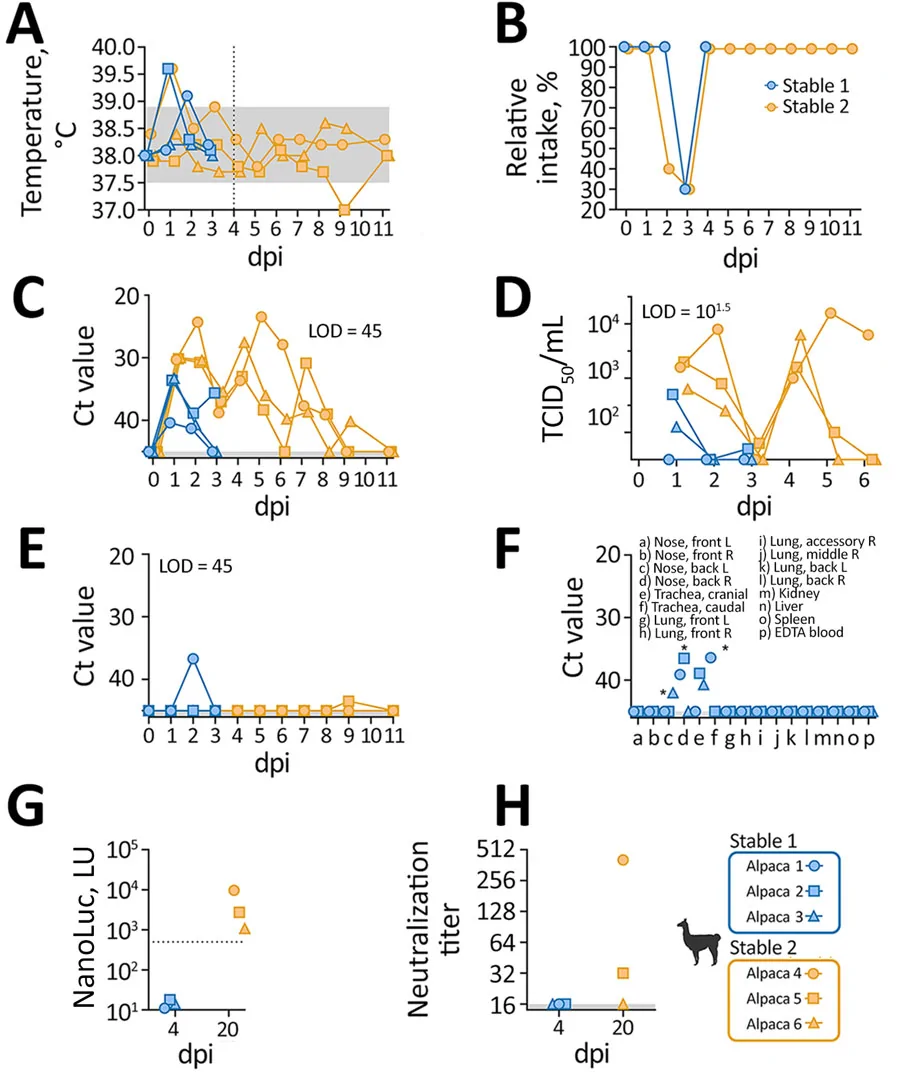
Clinical and laboratory findings from experimental highly pathogenic avian influenza A(H5N1) clade 2.3.4.4b virus infection in alpacas, 2026. Stable 1 alpacas were sampled before euthanasia at 4 dpi; stable 2 alpacas were sampled for 20 dpi before euthanasia. A, B) Clinical signs; C–H) laboratory findings. A) Body temperature; shading indicates physiologic range; dotted vertical line indicates dpi that stable 1 alpacas were euthanized. B) Percentage of typical feed intake. C) Quantitative reverse transcription PCR (qRT-PCR) results on nasal swab samples showing H5N1 viral genome was detected until 9 dpi. Shading indicates LOD. D) Nasal swab sample infectious titers. E) qRT-PCR results on rectal swab samples. Shading indicates LOD. F) qRT-PCR results on tissue samples. Of note, only nose and trachea samples were qRT-PCR–positive as evaluated by endpoint dilution assays for quantification of infectious virus. Asterisks indicate samples that tested positive for infectivity by endpoint dilution at 10^1.88^, 10^1.82^, and 10^1.62^ TCID_50_/mL. G) ELISA specific for H5 antibodies, confirming positivity of samples collected at 20 dpi. H) Neutralization titers determined by using a live virus neutralization test against 100 TCID_50_, with the endpoint defined as complete protection from cytopathic effect. Titers are expressed as reciprocal serum dilution, confirming measurable neutralizing capacity for 2 of the samples that tested positive by ELISA. dpi, days postinfection; L, left; LOD, limit of detection; LU, light unit; NanoLuc, nanoluciferase; R, right; TCID_50_, 50% tissue infective dose.

We retrospectively estimated the semiquantitative feed intake at the stable level. At 2 dpi, feed intake in stable 2 decreased to ≈40% of typical intake; at 3 dpi, both stables had feed intake of ≈30% of the typical intake (Figure 1, panel B). Feed intake in both stables returned to 100% at 4 dpi.

### Transient Respiratory Virus Shedding

Cycle threshold (Ct) values <45 reflect detection of viral RNA; we considered Ct values <30 to indicate high viral shedding. Nasal swab samples began yielding positive results at 1 dpi with a mean Ct value of 32.95 (Figure 1, panel C). Samples from animals 1 and 3 became negative at 3 dpi, 1 day before planned euthanasia, but other animals constantly shed viral RNA for several days. At 9 dpi, animal 6 was the last to shed traces of viral genome (Ct value 40.17). Two animals demonstrated high viral genome loads on various days: animal 6 at 1 dpi (Ct value 29.98) and 4 dpi (Ct value 27.56) and animal 4 at 2 dpi (Ct value 24.30), 5 dpi (23.47), and 6 dpi (27.90). At 11 dpi, all nasal swab samples tested negative. 

Nasal swab sample virus titers showed high individual variation: at 1 dpi, titers ranged from negative (below limit of detection [LOD], 10^1.5^ TCID_50_/mL) to 10^3.3^ TCID_50_/mL; at 4 dpi, titers were 10^3^ to 10^3.8^ TCID_50_/mL; and at 6 dpi, titers ranged from negative to 10^3.8^ TCID_50_/mL (Figure 1, panel D). Rectal swab samples did not demonstrate continuous viral shedding by qRT-PCR (Figure 1, panel E).

Among organ samples collected at 4 dpi from stable 1 animals, individual nose and trachea samples revealed slightly positive Ct values of 36.4–42 (LOD 45), all remaining organ samples tested negative (Figure 1, panel F); we observed the same for samples collected at 20 dpi (data not shown). Of note, the back right nose sample of animal 2 and the cranial trachea sample and back left nose sample of animal 1 tested positive for virus genome by PCR and by endpoint dilution at 10^1.88^, 10^1.82^, and 10^1.62^ TCID_50_/mL (Figure 1, panel F). 

We used MinION (Oxford Nanopore Technologies, https://nanoporetech.com) to conduct next-generation sequencing on washing samples collected at 6 dpi (Appendix). Those results did not indicate any mutations. We deposited sequences in GISAID (accession no. EPI_ISL_20453888). 

### Humoral Immune Response

All 20 dpi serum samples tested positive for H5 binding antibodies by the in-house ELISA, but samples from animals euthanized at 4 dpi were nonreactive (Figure 1, panel G). The H5-specific commercial ELISA confirmed those results for all but 1 animal (Appendix Figure 2). Of note, microneutralization confirmed the ELISA positivity in 2 of 3 animals at 20 dpi; the weakly ELISA-positive animal was negative by microneutralization (Figure 1, panel H).

### IHC and ISH 

IHC and ISH identified viral RNA and antigen in the nasopharyngeal tonsil from all 3 animals euthanized at 4 dpi. ISH showed marked labeling, but antigen detection was less prominent. In animals 1 and 3, virus detection was limited to cytoplasmic signals in macrophages or dendritic cells, indicating phagocytosis of virus RNA and protein (Figure 2, panels A–C). In alpaca 2, we detected virus RNA and minimal virus protein in the nucleus and cytoplasm in cells of the follicle-associated epithelium, indicating infection and potential replication (Figure 2, panels D–F). Only alpaca 1 exhibited viral RNA within the lumen of the left cranial nasal cavity, associated with cellular detritus (Figure 2, panel G).

**Figure 2:**
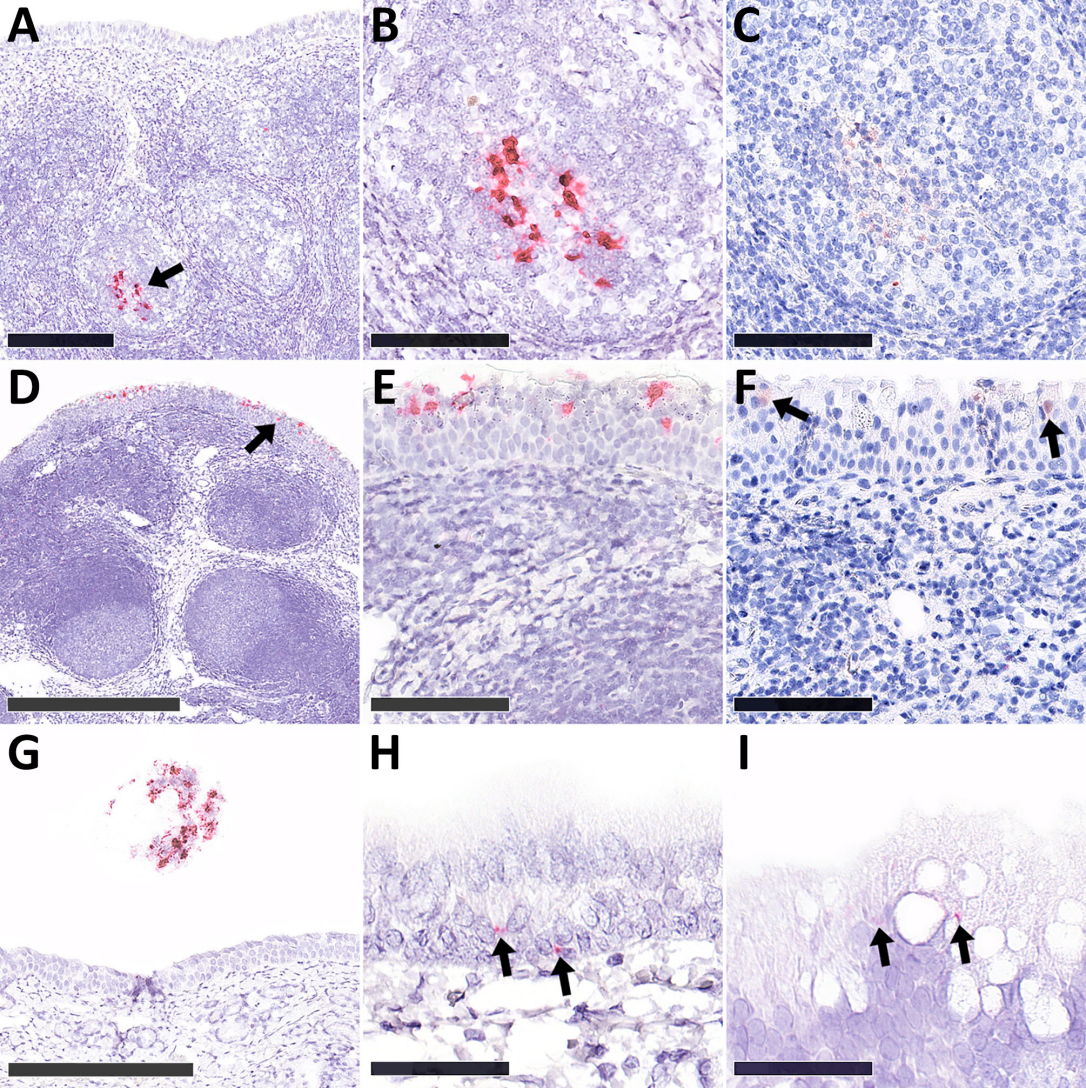
Histopathologic detection in nasopharyngeal tonsil and nasal conchae from experimental highly pathogenic avian influenza A(H5N1) clade 2.3.4.4b virus infection in alpacas, 2026. In situ hybridization (ISH) performed by using Fast Red chromogen; immunohistochemistry (IHC) performed by using 3-amino-9-ethylcarbazole substrate for red-brown chromogen; Mayer’s hematoxylin counterstain used for both ISH and IHC. A, B) ISH showing strong cytoplasmic RNA labeling (arrow in A [scale bar indicates 250 µm], detail in B [scale bar indicates 100 µm]) from nasopharyngeal tonsil of alpaca 3. C) IHC of nasopharyngeal tonsil from alpaca 3 showing weak antigen signals within follicular macrophages and dendritic cells; scale bar indicates 100 µm. D–F) Tonsillar epithelium from alpaca 2. D, E) ISH showing viral RNA (arrow in D [scale bar indicates 250 µm], detail in E [scale bar indicates 100 µm]); F) IHC showing antigen (arrows) in the nucleus and cytoplasm. Scale bar indicates 100 µm. G–I) ISH showing abundant detection of viral RNA within cellular debris on left cranial nasal cavity tissue from alpaca 1 (G; scale bar indicates 250 µm), nasal epithelium of alpaca 1 at 4 days postinfection (H), and alpaca 4 at 20 days postinfection (I) (arrows). Scale bars indicate 50 µm.

Whether the minimal viral RNA detected in the ciliated respiratory nasal epithelium in animals 1 and 4 (Figure 2, panels H, I) is attributable to infection remains speculative. Histopathology revealed minor lesions consistent with rhinitis (animals 1, 2, 4–6), tracheitis (animals 5, 6), and 1 instance each of chronic interstitial pneumonia (animal 1), pleuritis (animal 3), and even encephalitis affecting the olfactory bulb (animal 6). However, none of the findings can be reliably associated with IAV infection because we did not detect viral RNA nor viral antigen associated with those lesions by IHC and ISH (Appendix Table 1, Figure 1). In addition, brain paraffin sections of animal 6 tested PCR-negative for viral genome.

## Discussion

In May 2024, HPAI H5N1 clade 2.3.4.4b virus infection was confirmed in alpacas, the first documented natural infection of this virus type in camelids (*27*). The detection occurred on premises where poultry had been culled because of HPAIV H5N1, and genomic analysis revealed that the viral sequence was identical to the B3.13 genotype circulating in US dairy cattle and consistent with the depopulated poultry on site (*27*).

Camelid IAV infections have previously been reported. For instance, after the 1977 influenza epidemic in Russian, an epizootic was reported among camels in Mongolia (*35*). A reassorted virus was identified as the cause but was shown to be antigenically highly related to A/USSR/90/77 (H1N1), the strain from the epidemic in Russia (*35*). In the same region, camel infections with low pathogenicity avian influenza H7N9 and equine H3N8 viruses also have been reported (*36*,*37*). IAV exposure and infection, including H1 and H3 subtypes, have been detected in camels in Africa (*38*). However, H5 infection had not been reported in camelids until HPAIV H5N1 was confirmed in US alpacas in 2024 (*27*). Although transmission was not unexpected considering high viral load in the environment and co-mingling of livestock species (*27*), identification of alpacas as susceptible hosts expands the known mammalian host range of HPAIV H5N1. The experimentally generated data from this study, including the detailed pathologic characterization, will enable meaningful comparisons in the future.

Roughly 262,000 alpacas are housed in the United States (*39*). In South America, the total camelid population is much larger, ≈12 million (*40*). Although no H5N1 infections have been reported among camelid species in South America, the large camelid population, comprising ≈4.0 million llamas and ≈7.5 million alpacas in domestic herds, and ≈350,000 vicuñas and ≈600,000 guanacos in wild herds, represents a substantial interface with humans and a huge potential reservoir (*40*). Among domesticated species, 85% are raised by small producers, often women, with herds of <100 animals playing a fundamental role in milk, meat, and fiber production (*40*). That demographic and livestock structure underscores why surveillance in a diverse set of mammalian hosts is vital for public health, especially considering H5N1 clade 2.3.4.4b virus has caused mass die-offs in sea lions and elephant seals in South America since late 2022 (*41*). That marine mammal–adapted virus clade has shown mutations consistent with mammal-to-mammal transmission, raising concern for further mammalian adaptation (*41*). 

H5N1 clade 2.3.4.4b viruses in North America comprise multiple genotypes and efficiently replicate and spread in wild bird populations (*42*). Those factors increase risk for clade 2.3.4.4b virus spillover to a range of mammals, including ruminants, carnivores, and camelids, potentially enhancing the virus’ capacity for adaptation to mammalian hosts. Most bird-to-mammal transmissions appear to be dead-end infections mostly affecting terrestrial carnivores feeding on infected prey (*19*). Mammal-to-mammal transmissions rarely become established because large mammal groups and frequent contact are needed to maintain transmission chains; however, H5N1 receptor specificity remains avian α2-3–like (*43*). That observation matches the observed clinical picture for bovine and zoonotic infections because α2-3 linked sialic acids are abundant not only in avian species but also in bovine mammary glands and human conjunctiva (*11*). Nevertheless, efficient mammal-to-mammal H5 virus transmission has been confirmed in mink farms (*44*), among aquatic mammals in South America (*41*,*45*), and from cattle to cats and humans via milk (*20*). Those transmission events correlated with accumulation of further mammal-adaptive mutations (*11*,*46*), and a shift toward mammal-adapted variants was evident in some cases, reflected in accumulation of multiple mammalian-adaptive mutations (*11*). Data of intramammary and oronasal experimental H5N1 B3.13 virus infection of goats show efficient virus replication in the mammary glands, comparable to that in cows, but only limited virus replication and necrotizing interstitial pneumonia at early (3 dpi) timepoints (*47*).

We experimentally confirmed that alpacas can be intranasally infected with a bovine HPAIV H5N1 B3.13 isolate, leading to subsequent nasal shedding of infectious virus. IHC and ISH confirmed H5N1 virus replication in the nasopharyngeal tonsil and the nasal conchae. Productive infection was corroborated by seroconversion at 20 dpi. We detected individual variation of neutralizing antibody levels, but the small number of animals does not enable general conclusions. The infection did not cause fever, but alpacas reduced feed intake. Whether the viral load shed by the alpacas would be sufficient to cause transmission to contact animals remains undetermined. Intermittent viral RNA detection and low viral titer at 3 dpi might reflect technical variability of the sampling procedure rather than reduced shedding. 

Our data did not suggest that alpacas replicate H5N1 clade 2.3.4.4b genotype B3.13 virus with exceptionally high efficiency. However, nasal replication occurred, and infectious virus was detectable in nasal swab samples for up to 6 dpi, but sequencing did not indicate accumulation of mutation to that timepoint. Nevertheless, viral adaptation after infection or shedding to naive contact animals or humans cannot be excluded. A follow-up study should include direct-contact animals to assess the biologic relevance of low-level shedding and to clarify the potential role of alpacas in H5N1 transmission. In addition, future studies should include experimental intramammary inoculation of female alpacas to evaluate susceptibility via that route. 

In conclusion, H5N1 clade 2.3.4.4b virus will likely continue to drive substantial mortality rates in wild birds and marine mammals across North and South America. Risk for virus spillover into domestic animals, including poultry and potentially camelids, will continue considering their large populations on the continents. The demonstrated capacity of H5N1 clade 2.3.4.4b virus to acquire mammalian-adaptive mutations heightens concern about cross-species transmission and possible establishment in new mammalian hosts, which could intensify wildlife losses, disrupt livestock production, and generate new zoonotic risks. Sustained surveillance, strengthened biosecurity, and rapid response measures will be essential to limit those impacts.

Appendix Additional information on experimental highly pathogenic avian influenza A(H5N1) clade 2.3.4.4b virus infection in alpacas, 2026.
