# Supplementary material for: Experimental Highly Pathogenic Avian Influenza A(H5N1) Clade 2.3.4.4b Virus Infection in Alpacas, 2026
Source: Emerg Infect Dis. 2026 Oct;32(10):1657–66. doi: 10.3201/eid3210.260491 (PMC13632876; doi:10.3201/eid3210.260491)
Supplement: Supplementary file 1 [file 26-0491-Techapp-s1.pdf]

Article DOI: https://doi.org/10.3201/eid3209.260491

EID cannot ensure accessibility for supplementary materials supplied by authors. Readers who have difficulty accessing supplementary content should contact the authors for assistance.

Experimental Highly Pathogenic Avian Influenza A(H5N1) Clade 2.3.4.4b Virus Infection in Alpacas, 2026

Appendix

Additional Methods

MinION-based sequencing

As described earlier (*1*,*2*) the RNA of influenza positive samples was extracted using the NucleoMag® VET Kit (#744200.4, Machery-Nagel GmbH & Co. KG, Düren, Germany) on the KingFisher platform. Sequencing of avian influenza-positive samples collected in Germany 2024–2025 was performed using an amplicon-based protocol on nanopore platforms (Oxford Nanopore Technology, Oxford, UK). Briefly, RNA was transcribed into DNA using the Superscript III One-Step and Platinum Taq kit (Thermo Fisher Scientific, USA) with Influenza A specific primers, each binding to the conserved 3′ or 5′ end of all Influenza RNA segments (Pan-IVA-1F_BsmF: TATTCGTCTCAGGG-AGCRAAAGCAGG; Pan-IVA-1R_BsmR: ATATCGTCTCGTATT-AGTAGAAACAAGG). DNA amplicons were purified with Agencourt AMPure XP magnetic beads (Beckmann Coulter, Krefeld, Germany) using DNA LoBind Tubes (Eppendorf, Wesseling-Berzdorf, Germany). Quantification of nucleic acids was done with Qubit Fluorometry (Thermo Fisher Scientific, USA). Approximately 200 ng of cDNA was sequenced using a transposase-based library preparation approach with Rapid Barcoding (SQK-RBK114, Oxford Nanopore Technologies, Oxford, UK) and on a PromethION P2 Solo instrument with the latest MinKNOW software core (v6.5.14). High accuracy base calling of the raw data using Dorado (v7.9.8, Oxford Nanopore Technologies) was followed by demultiplexing, a quality check and a trimming step to remove poor quality, adaptor and short (<20 bp) sequences. The generated data were stored in FASTQ and POD5 data formats. The

bioinformatics software suite Geneious Prime (GraphPad Software LLC, version 2025.1.3) was used for analysis. Sequences were trimmed to remove primer sequences. Consensus sequences were obtained using an iterative map-to-reference approach with Minimap2 (vs 2.24) (*3*). Reference genomes were selected from a curated collection of all HA and NA subtypes and a selection of internal gene sequences to cover all potentially circulating viral strains. Polishing of the final genome sequences and annotation was performed manually after consensus generation (threshold matching 60% of bases of total adjusted quality). The consensus sequences are available under the accession numbers: EPI_ISL_20453888.

References

1. Ahrens AK, Jónsson SR, Svansson V, Brugger B, Beer M, Harder TC, et al. Iceland: an underestimated hub for the spread of high-pathogenicity avian influenza viruses in the North Atlantic. J Gen Virol. 2024;105:001985. PubMed https://doi.org/10.1099/jgv.0.001985

2. Halwe NJ, Cool K, Breithaupt A, Schön J, Trujillo JD, Nooruzzaman M, et al. H5N1 clade 2.3.4.4b dynamics in experimentally infected calves and cows. Nature. 2025;637:903–12. PubMed https://doi.org/10.1038/s41586-024-08063-y

3. Li H. New strategies to improve minimap2 alignment accuracy. Bioinformatics. 2021;37:4572–4. PubMed https://doi.org/10.1093/bioinformatics/btab705

Appendix Table. Pathology data (macroscopy and histopathology)

Measure	Details	Findings					
Animal ID		1	2	3	4	5	6
dpi		4	4	4	20	20	20
Macroscopy							
Lung	alveolar edema, acute, diffuse	mild	mild	mild	mild	mild	mild
	congestion, acute, diffuse	/	moderate	minimal	severe	severe	moderate
Histopathology							
conchae, all samples	intraluminal cellular debris with viable/degenerate neutrophils, protein-rich mucus	/	/	present	present	present	/
conchae, respiratory, right	submucosal, lymphoplasmacytic infiltrates, mucosal immune cell transmigration, no edema, no vascular activation (present = still within normal limits)	/	present	present	present	present	/
	rhinitis, with submucosal, lymphoplasmacytic infiltration, fewer neutrophils, mucosal immune cell transmigration, epithelial degeneration/necrosis, endothelial activation	/	/	/	minimal	/	/

Measure	Details	Findings					
conchae, respiratory, left	submucosal, lymphoplasmacytic infiltrates, minimal mucosal immune cell transmigration, no edema, no vascular activation (present = still within normal limits)	present	present	present	present	present	present
	rhinitis, with submucosal, lymphoplasmacytic infiltration, fewer neutrophils, mucosal immune cell transmigration, epithelial degeneration/necrosis, endothelial activation	/	/	/	mild	minimal	minimal
conchae, olfactory, right	submucosal, lymphoplasmacytic infiltrates, minimal mucosal immune cell transmigration, no edema, no vascular activation (present = still within normal limits)	/	/	/	/	present	present
	rhinitis, with submucosal, lymphoplasmacytic infiltration, fewer neutrophils, mucosal immune cell transmigration, epithelial degeneration/necrosis, endothelial activation	minimal	/	/	/	/	moderate
conchae, olfactory, left	submucosal, lymphoplasmacytic infiltrates, minimal mucosal immune cell transmigration, no edema, no vascular activation (present = still within normal limits)	/	/	/	present	present	/
	rhinitis, with submucosal, lymphoplasmacytic infiltration, fewer neutrophils, mucosal immune cell transmigration, epithelial degeneration/necrosis, endothelial activation	minimal	minimal	/	mild	/	/
tonsil, nasopharyngeal	tonsillitis, with increased number of tingible body macrophages in follicles, multifocal immune cell transmigration with epithelial degeneration, edema	mild	mild	mild	minimal	minimal	moderate
lymph node, mandibular	lymphadenitis, with increased number of tingible body macrophages, prominent sinus macrophages, few neutrophils, edema	mild	/	minimal	minimal	minimal	mild
lymph node, tracheobronchial (left)	no finding	/	/	/	/	/	/
trachea, cranial	submucosal, lymphoplasmacytic infiltrates, mucosal immune cell transmigration, no edema, no vascular	/	/	present	present	present	present

Measure	Details	Findings					
	activation (present = within normal limits)						
	tracheitis, with submucosal, lymphocytic infiltration, few neutrophils, mucosal immune cell transmigration, epithelial degeneration/necrosis, endothelial activation	/	/	/	/	minimal	/
trachea, caudal	submucosal, lymphoplasmacytic infiltrates, mucosal immune cell transmigration, no edema, no vascular activation (present = within normal limits)	present	/	/	present	/	present
	tracheitis, with submucosal, mononuclear infiltration, few neutrophils, mucosal immune cell transmigration, epithelial degeneration/necrosis, endothelial activation	/	/	/	/	/	minimal
lung	congestion, acute	severe	severe	moderate	moderate	severe	severe
	edema, alveolar, acute	/	moderate	minimal	severe	severe	moderate
	BALT prominent	present	present	present	present	/	present
	granuloma, alveolar, focal	present	/	present	/	present	present
	fibrosis, pleura	/	/	present	/	present	/
lung, right, middle	pleuritis, chronic-active, granulomatous and eosinophilic, focal-extending	/	/	moderate	/	/	/
lung, left, cranial	pneumonia, chronic, interstitial, with follicle/GC formation and pleural fibrosis;	moderate	/	/	/	/	/
olfactory bulb	infiltrates, multifocal, lymphoplasmacytic, with endothelial activation, grade	/	/	/	/	/	moderate
brain, cortex	no finding	/	/	/	/	/	/
hippocampus	no finding	np	np	/	/	/	/
brain stem	no finding	/	/	/	/	/	/
cerebellum	no finding	/	/	/	/	/	/

*np, not present on slide.

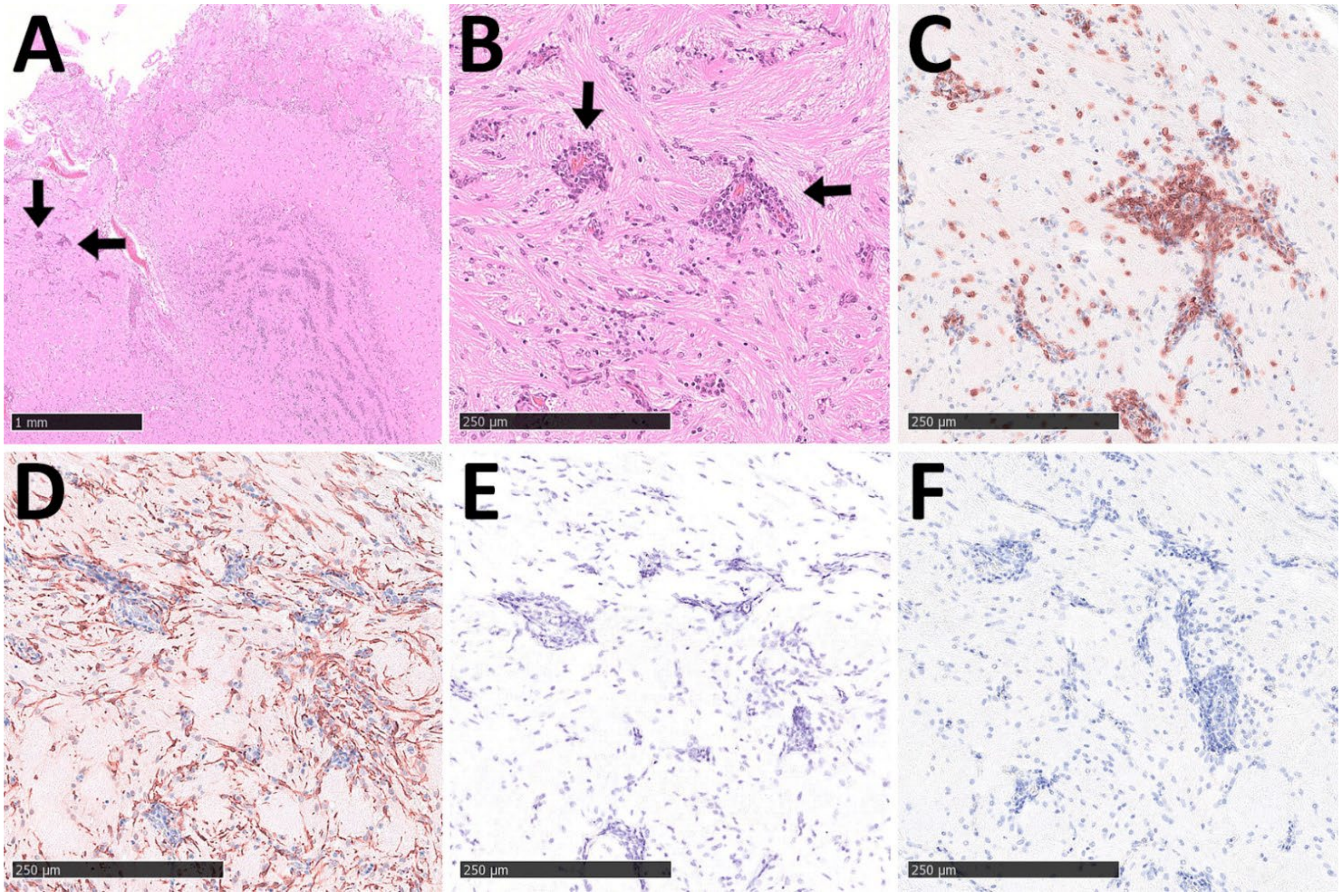

Appendix Figure 1. Encephalitis in the olfactory bulb of alpaca no. 6, 20 days after HPAIV H5N1 infection, without virus RNA and virus protein detection. (a) Encephalitis in the olfactory bulb with prominent perivascular infiltrates (hematoxylin-eosin staining), highlighted with arrows and shown in detail in (b), mostly comprised (c) CD3 positive T-lymphocytes (immunohistochemistry, IHC, anti-CD3). (d) Presence of numerous reactive Iba-1 positive microglia/macrophages (IHC, anti-Iba-1). (e) No detection of IAV RNA by RNA in situ hybridization or (f) IAV antigen by IHC (anti-IAV nucleoprotein). Bar (a) 1 mm (b-f) 250µm.

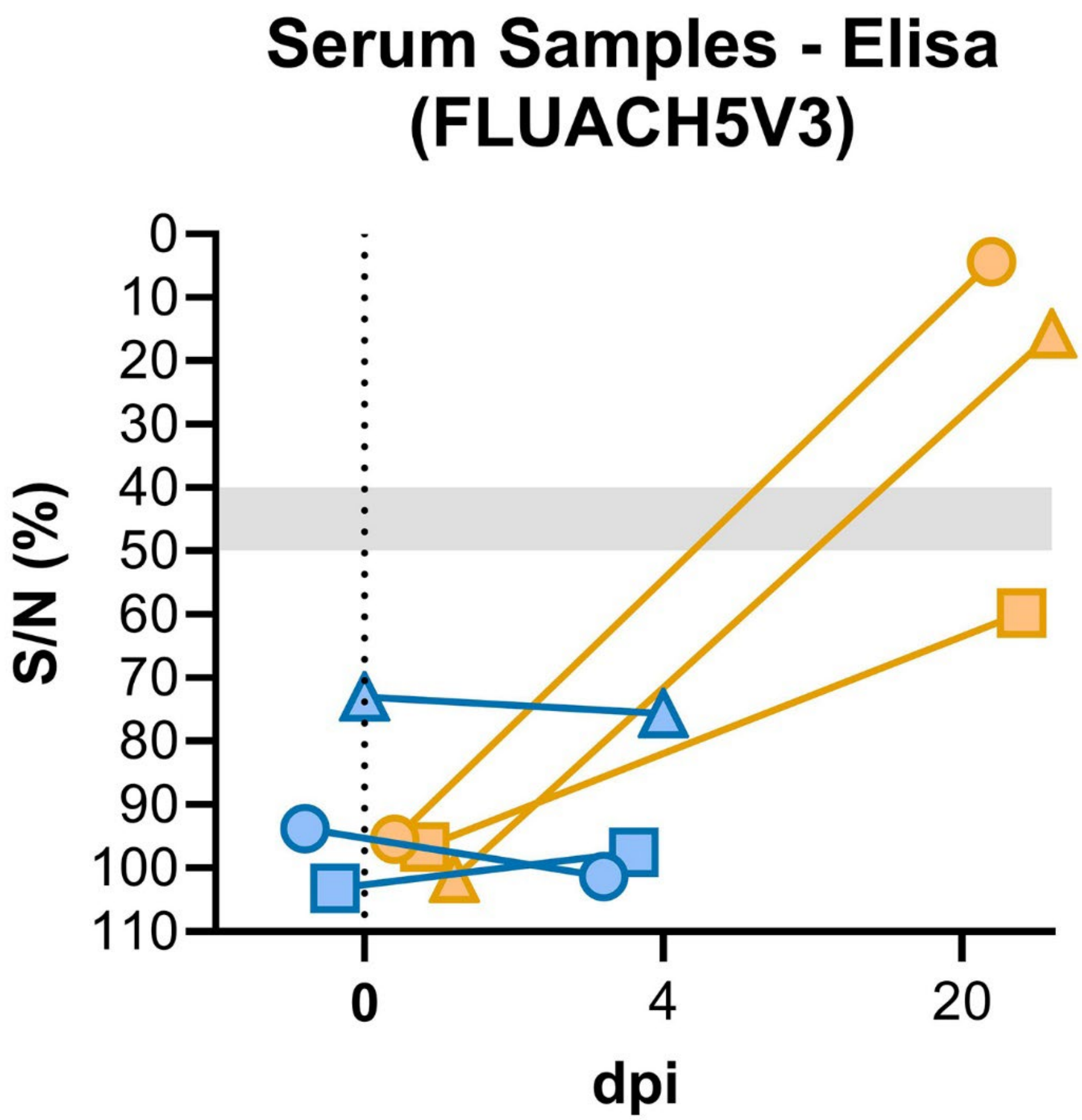

Appendix Figure 2. Humoral immune response following H5N1 infection in alpacas evaluated by commercial H5-specific ELISA. Using the adapted H5-ELISA (FLUACH5V3) two out of three sera of 20 dpi have been clearly tested positive and the third was near the applied threshold (for bovine samples).
